# The Effect of Conformation Order on Gas Separation Properties of Polyetherimide Ultem Films

**DOI:** 10.3390/polym12071578

**Published:** 2020-07-16

**Authors:** Julia Kostina, Sergey Legkov, Alexander Kolbeshin, Roman Nikiforov, Denis Bezgin, Alexander Yu. Nikolaev, Alexander Yu. Alentiev

**Affiliations:** 1 A.V. Topchiev Institute of Petrochemical Synthesis Russian Academy of Sciences, 119991 Moscow, Russia; legkov@ips.ac.ru (S.L.); kolbas@ips.ac.ru (A.K.); nru@ips.ac.ru (R.N.); bezgin@ips.ac.ru (D.B.); 2 A.N. Nesmeyanov Institute of Organoelement Compounds of Russian Academy of Sciences, 119334 Moscow, Russia; nikolaev@polly.phys.msu.ru

**Keywords:** gas separation, polyetherimide, FTIR spectroscopy, conformational order, quantum chemical calculations, non-covalent interactions, residual solvent

## Abstract

Changes of the spectral characteristics of absorption bands depending on the films’ treatment method were registered for polyetherimide Ultem films. The possibility of selection of structural criteria (the ratio of the functional groups absorption bands intensities) showing all conformational changes in elementary unit with metrological processing of the results is shown. It is demonstrated that film formation from chloroform solution leads to elementary unit fragments, Ph–O–Ph′, which have an effect on macromolecule conformation and result in increasing of space between fragments of macromolecules (local polymer matrix packing loosening). Desorption of residual chloroform from films by ethanol or supercritical CO_2_ leads to a change of conformers set in Im–Ph–Im′ units. Quantum chemical modeling showed the possibility of convergence of these fragments in neighboring macromolecules, and consequently of interchain π–π interaction (local densification of chain packing of the polymer matrix). After annealing at a temperature higher than glass transition temperature, the polyetherimide film exhibits the most disordered (amorphous) state at all of the fragments. It is demonstrated that the results, obtained by the combination of theoretical and experimental vibrational spectroscopy methods, are in good agreement with data of chain packing ordering found by analysis of gas separation parameters.

## 1. Introduction

One of the key problems of the application of glassy amorphous polymers is a dependence of sample properties and its history. This is due to the thermodynamic nonequilibrium of the glassy state. Therefore, glassy amorphous polymers are used in different stationary states which depend on preparation technique and operation mode. Especially, significant effects occur for thin films and coatings. For example, glassy amorphous polymers are applied as materials for thin continuous layers (several tens up to several hundred nanometers) of nonporous gas separation membranes. Therefore, specifically for membrane material science, the stability of gas separation properties in time and the effect of preparation technique and operation mode of the polymer membrane (film) on its gas separation characteristics are very important issues [1]. As a rule, polymer membranes are made out of polymer solutions, thus gas separation properties are sensitive to solvent type and polymer solution concentration [2,3,4,5], presence of residual solvent, and method of its removal [6,7,8,9]. Thermodynamic nonequilibrium state of glassy amorphous polymers leads to a change in nonequilibrium free volume and consequently gas separation properties of polymer membranes during exploitation due to physical aging [1,9,10] or plasticization with a separated component [1,11]. On the other hand, this is why gas separation characteristics might be considered as a nonequilibrium level assessment criterion for polymer film, prepared by one method or another [12]. As there are no direct methods of measurement for chain packing ordering for X-ray amorphous polymers, a combination of analysis of gas diffusion coefficients [12] with the results of other methods can be promising for the determination of a trend for a change of chain packing ordering and the effect of this trend on physicochemical and transport properties. One of these methods is vibrational spectroscopy, which provides evaluation of conformational structure change on atomic level and molecular level. An additional factor supporting the selection of vibrational spectroscopy as independent method of assessment of tendency of treatment technique effect on conformational structure of X-ray amorphous polymers is a well-developed theory of vibrational spectroscopy. It allows one to use both quantum chemistry methods to interpret experimental data and alternatively to substantiate reliability of theoretical calculations by results generated by test samples IR spectra analysis. Using a combination of experimental and theoretical methods of vibrational spectroscopy, and based on the results of an analysis of changes of test samples spectra, it is now also possible to resolve the inverse problem of vibrational spectroscopy for polymers, i.e., to register changes of elementary unit structure based on the model justified by experimental spectral data. This approach can be successfully applied to calculate interactions, including noncovalent interactions. Comparison of theoretical vibrational spectra with experimental ones can allow one to make adequate conclusions and to find relationship between structural (particularly, conformational) changes in macromolecule and polymeric object properties altogether.

Previously, in [8,13,14,15,16], it has been shown that interaction between functional groups of polymer with a solvent with formation of hydrogen bonds leads to reorientation of these groups over the polymer backbone, changing its conformational parameters [13,14,15,16]. If hydrogen bond formation leads to an increase in the quantity of elementary unit conformers of the same geometry, it is appropriate to suppose ordering of conformational composition of elementary units of macromolecule, which results in chain packing ordering. In turn, polymer chain packing ordering may lead to modification of free volume elements distribution in the matrix [15]. In these cases, the modes ratio in bimodal free volume elements (FVE) size distribution changes with an increase of specific mode concentration. Such modification of FVE size distribution is accompanied by a sharp increase in gas separation selectivity with maintaining [13,15] of gas permeability (sometimes these data exceed upper bound [15]) of permeability-selectivity diagram [17], which makes polymer material perspective for membrane gas separation. In [15], the relationship of gas separation parameters with conformational chain ordering when the polymer is treated with non-solvents (alcohols) also forming complexes with hydrogen bonds was also considered.

Gas separation selectivity growth with gas permeability increase has also been observed for polyetherimides (PEI) films swelling in supercritical CO_2_ (*sc*-CO_2_) with further slow depressurization (0.008–0.025 MPa/min) [18,19,20]. It is well known that polymer treatment with *sc*-CO_2_ facilitates desorption of a residual solvent [21] independently of its capability to form hydrogen bonds with polymer functional groups. However, according to the authors of [12], such film treatment can lead to increase of chain packing ordering in polyetherimides. Nevertheless, there has been no confirmation of changes of conformational chain ordering after amorphous polymer film treatment with *sc*-CO_2_ obtained by other methods.

The aim of this work is to verify the hypothesis on the effect of different film formation and treatment conditions on conformational order of macromolecules.

**Scheme 1:**
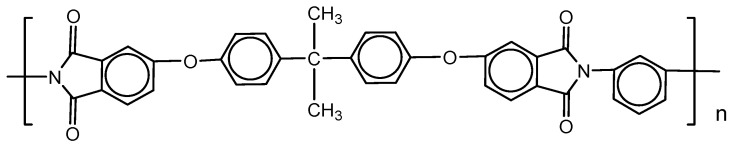
Elementary unit of Ultem structure (*T*_g_ = 215 °C).

Quantitative criteria of amorphous polymer chain packing ordering are proposed for PEI Ultem (Scheme 1) based on analysis of structural characteristics from IR spectra of polymeric films at different formation and treatment conditions and diffusion coefficients of non-condensable gases (H_2_, He, N_2_, O_2_, CO_2_, and CH_4_). Formation of hydrogen bonds has been noted earlier for this polymer [13,15], and quantitation of chain packing ordering change has been performed using gas separation characteristics analysis [12] at different film formation and treatment conditions.

## 2. Materials and Methods

### 2.1. Film Formation

All Ultem PEI films (ULTEM^®^ 1000 Resin, SABIC Innovative Plastics, Riyadh, Saudi Arabia) were formed from a 5% solution in chloroform (ChP).

For gas separation properties investigation, films of 30–35 µm thickness were prepared by casting over a cellophane support from 5% solution in chloroform with subsequent drying for 2–3 days at ambient temperature and then keeping them in vacuum at 1–2 mmHg until constant weight is achieved.

Films for investigation by vibrational spectroscopy with a thickness of 5–7 μm were formed from a solution of chloroform on optical glass made of silicon and dried at room temperature to constant weight. At least 2 films were formed for each state.

### 2.2. Film Treatment Methods

For gas separation experiments:

- As-cast film formation by solvent evaporation (see above); 12% to 13% of residual chloroform is fixed in films in these conditions [22];
- Annealing at 10–20 °C above the glass transition temperature [12,22];
- C_2_H_5_OH treatment—swelling in ethanol for one day with further evacuation [12,19];
- *sc*-CO_2_ treatment—swelling in *sc*-CO_2_ for 4 h at 120 °C and 450 atm.

For IR spectroscopy, film treatment on Si optical glasses repeated film treatment modes for gas separation films.

### 2.3. Investigation Methods

FTIR spectra of films on Si optical glasses were recorded on an IFS 66 v/s vacuum Fourier transform spectrometer (“Bruker”, Bremen, Germany, range: 400–4000 cm^−1^, resolution 1 cm^−1^). For metrological processing of the results, the IR spectra of each film were recorded at least twice, moving the sample in the cell compartment.

Polymer films density (ρ) was determined at room temperature 24 ± 2 °C by hydrostatic weighing in isopropanol. Fractional free volume (FFV) was determined by Bondi method: FFV = 1–1.3 V_w_/V_sp_, where V_w_ is Van der Waals volume of elementary unit, V_sp_ = M/ρ is a specific occupied volume of polymer, and M-polymer elementary unit molecular weight.

Diffusion coefficients (*D*) of N_2_, O_2_, CO_2_, and CH_4_ in films were obtained by integral barometric method on MKS Barotron assembly.

For metrological processing of the results, the average value of the relative optical density for the selected pairs of absorption bands $\bar{x}$ (*x = A*_1_*/A*_2_), the variance of the mean $V\left(x\right)=s^{2\;}\left(x\right)=\frac{\sum_{i=1}^{n}{\left(x_{i}-\bar{x}\right)}^{2}}{n-1}$, the relative standard deviation from the mean (reproducibility) $s_{r}\left(x\right)=\frac{s\left(x\right)}{\bar{x}}\;$ and the confidence interval ∆*A* were calculated.

The significance of the differences in the results for the series of films subjected to various treatment methods was evaluated using the modified Student’s test: the average standard deviation of two measurement series was found $\bar{s}(x)$ = $\sqrt{\frac{f_{1\;}V_{1}+f_{2\;}V_{2}}{f_{1}+f_{2}}}$ and test statistics *ξ* = $\frac{\left|\bar{x_{1}}-\bar{x_{2}}\right|}{\bar{s}\left(x\right)}\sqrt{\frac{n_{1}\cdot n_{2}}{n_{1}+n_{2}}}$, where *f*_1_ и *f*_2_—are the degrees of freedom for two series of measurement and *n*_1_ и *n*_2_—are the number of parallel measurements from which $\bar{x_{1}}$ и $\bar{x_{2}}$ were calculated; confidence probability *P* = 0.90.

Changes in structural characteristics were considered significant according to the condition for assessing the significance of the results for homogeneous dispersions, *ξ > t (P*, *f)*, where *t* is the Student’s coefficient for confidence probability *P* and the number of degrees of freedom *f*. For *ξ < t (P*, *f)* the changes were considered insignificant and it was assumed that the treatment method does not affect the conformational order in macromolecules or the sensitivity of the method is not sufficient for reliable recording of such effect.

## 3. Results and Discussion

### 3.1. Packing Ordering by Gas Separation Data

Similarly to slow depressurization Ultem films treatment with *sc*-CO_2_ with further fast depressurization [12,18] significantly decreases density and increases free volume (Table 1). However, similarly to slow depressurization case [12,18], free volume increase is not accompanied by diffusion coefficients increase: D is shown to decrease for “as cast” films (Table 1) and to practically remain the same for annealed films. At the same time, *sc*-CO_2_ treatment leads to significant diffusion selectivity increase in all cases (Table 1), which indicates chain packing ordering increase [12]. A similar effect, although less pronounced, is caused by film treatment with ethanol.

In [12], quantitative characteristics of packing ordering determined through parameters of linear regressions of gas diffusion coefficients and gas-kinetic diameters [23] are proposed (Table 1).

log *D* = *a* − *b*·*d*^2^ (1)

These characteristics are relative (compared to the closest to equilibrium state annealed sample) cohesion energy changes Δ*E_coh_* (inter-chain contacts in dense polymer matrix part quantity), entropy change of the basic state Δ*S*^0^ (Δ*S*^0^ increase is responsible for a chain packing ordering) and, finally, experimental value of diffusion selectivity α*^D^* (higher diffusion selectivity means that chain fragment packing in dense polymer matrix part is denser, more ordered).

According to these criteria, the most disordered is the packing of chains in the annealed sample. (Table 1). The unannealed “as-cast” sample shows higher level of order, which was previously explained by elementary unit conformational composition order resulting from non-covalent bonding of oxygen (O) atom in phenyl-ether fragment with chloroform [13,14,15,16]. Ethanol and *sc*-CO_2_ treatment leads to further ordering. In this case, *sc*-CO_2_ treatment effect on ordering depends on depressurization rate. *sc*-CO_2_ treatment with slow depressurization leads to the greatest ordering. Film samples prepared by *sc*-CO_2_ treatment with fast depressurization have lower density and higher free volume, but its ordering levels are comparable to the samples prepared by ethanol treatment (Table 1).

### 3.2. Conformation Order of Films According to Quantum Chemical Modeling Data

The previously considered interaction of polymer functional groups with a solvent, and as a result formation of hydrogen bonds, can lead to a reorientation of polymer functional groups about a polymer backbone changing its conformational parameters and increasing the content of a conformer of a single geometry (conformation order) [13,14,15,16]. Relevant effects of such conformation order are possible only with a particular value of comparator parameter for kinetic chain rigidity. The authors suggested using the proportion of phenyl-ether junctions per elementary unit (*k*), which was calculated as the ratio of the number of ether O atoms to the number of phenyl rings in the PI backbone as such parameter. For Ultem *k* = 0.4, whereas *meta*-substituted aromatic ring is located between the imide cycles, and aromatic cycles, and isopropylidene groups form a tetrahedron with which the *para*-substituted phenyl rings are connected. For such PI, despite the possibility of non-covalent interactions of Ph–O–Ph′ fragment with the solvent, conformation order in the elementary unit either cannot be achieved at all or is determined by other structural parameters. Therefore, the next task was to find a structural parameter (criterion) that (a) can be reliably determined from experimental spectral data, (b) uniquely characterizes changes of the conformational set of the elementary unit, and (c) can be compared with the measured or calculated physicochemical characteristics of a polymer object.

Specific inter-chain interactions leading to the creation of ordered regions and in the extreme—to a semicrystalline structure—for PI, which has only *para*-substituted aromatic cycles in the elementary unit at Ph–O–Ph′ fragments (Kapton) are described previously. A method for selecting an optimal conformation of the macromolecule in the crystalline region was developed for it, and variants of polymer chains packing were calculated [24,25]. In these works, it was shown that the *para*-substituted aromatic rings at the Ph–O–Ph′ nodes in Kapton are “hinges” that are characterized by a variety of conformers. It is noted that for any conformations of such fragments defined only by the angle of rotation, the length of the monomeric unit does not change. In this case, imide cycles form two types of conformations: “propeller” and “roof-like”, and the energy of these states is such that the transitions of one conformation to another are impossible, both exist simultaneously. This structure of PI leads to the possibility of intermolecular π–π interactions and formation of a denser packing of polymer chains.

The possibility of *π*–*π* interaction for fragmentary models of Ultem containing an aromatic ring between imide cycles (Im–Ph–Im′ fragment) was evaluated by calculation of geometric, energy, and electronic parameters (HF/6-31G(d, p)) and bringing the calculations to the theoretical vibrational spectra (B3LYP/6-31G(d, p)).

Quantum chemical calculations have shown an almost equal probability of the existence of two conformers of Im–Ph–Im′ fragment (Table 2) with different values of geometric parameters. In other words, similar to the “propeller” and “roof-like” conformation in Kapton, in Ultem, too, both Im–Ph–Im′ conformations exist simultaneously, and inter-chain *π*–*π* interaction between these polyheteroarylene fragments bound by a *meta*-substituted aromatic ring becomes possible.

Calculations of the geometric parameters of two fragments located relative to each other in a “roof-like” conformation (model III) confirmed our assumption: the interaction energy (∆E ~−16.4 kJ/mol, Table 2), the distance between the atoms and the change in charges on the atoms correspond to π–π interaction between Im–Ph–Im′ fragments. For example, the change in charge on the oxygen atoms of the imide cycle in absolute value is |∆q| ≈ 0.068–0.100, on nitrogen atoms ≈ 0.225–0.259; the change in bond lengths is ~2%.

Considering that the only *meta*-substituted aromatic ring is located between imide fragments in the elementary unit of PI, *π*–*π* interaction or its absence will, in the first place, affect polarization of C=O and C-N bonds in imide cycle, and, consequently, the change in optical density of absorption bands of ν_C=O_, ν_C–N_ stretching vibrations, and δ_NCO_, δ_CNC_ deformational oscillations. In addition, the effect should be noticeable on the deformational oscillations of protons (out of the plane of the ring) in the *meta*-substituted benzene ring in particularly. To accurately assign the absorption bands of the spectrum to the vibration types for models I and II (Table 2) theoretical vibrational spectra were calculated. Their analysis showed that the

- change of position of the *meta*-substituted aromatic ring relative to the imide cycles leads to an insignificant change in the frequency of the corresponding vibration in the theoretical vibrational spectrum (2–3 cm^−1^), while the spectra of both models are not identical;
- only 730 and 780 cm^−1^ frequencies refer to δ_CCH_ and δ_CNC_ vibrations, respectively.

**Figure 1:**
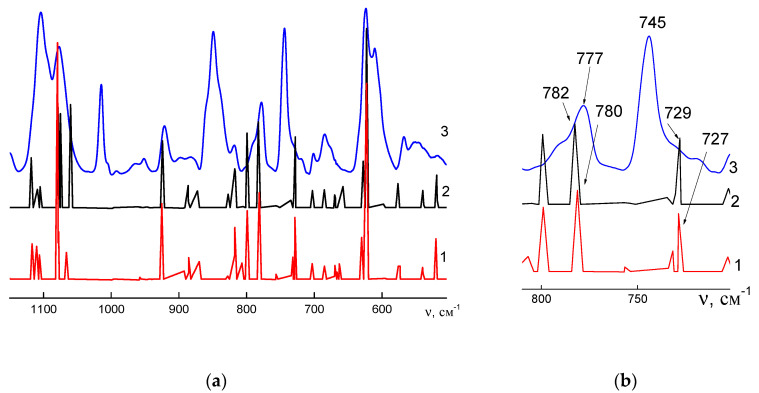
Fragments of theoretical vibrational spectra of models I and II of Im–Ph–Im′ unit in the region of 1050–500 cm^−1^ (**a**) and the absorption of the characteristic frequencies of the models (**b**), curves *1* and *2*, and a fragment of the IR spectrum of the Ultem film (*curve 3*).

In the experimental Ultem spectrum these frequencies correspond to absorption bands with maxima at 745 and 777 cm^−1^, which, given the limitations of the model in comparison with the PI macromolecule, can be considered a satisfactory result (Figure 1).

Summing up the analysis of the results of theoretical calculations, it can be noted that the π–π interaction or its absence in Ultem is possible only in Im–Ph–Im′ fragment, which should be reflected in all the absorption bands of the respective functional groups, though will not lead to a significant change in the positions of the maximum of the absorption bands for corresponding vibrations, but only to change of bonds polarization, i.e., to the change in intensity.

### 3.3. FTIR Spectroscopy

Thin (≤7 μm) Ultem films were cast from chloroform solution (as cast films) on an optical Si-glass. These films were (a) treated with ethanol, (b) treated with supercritical fluid (*sc*-CO_2_), (c) annealed, and (d) annealed and treated with *sc*-CO_2_. Minimum two films were formed for each treatment type. For further statistical processing IR-spectra of each film were registered in transmission mode not less than twice.

**Figure 2:**
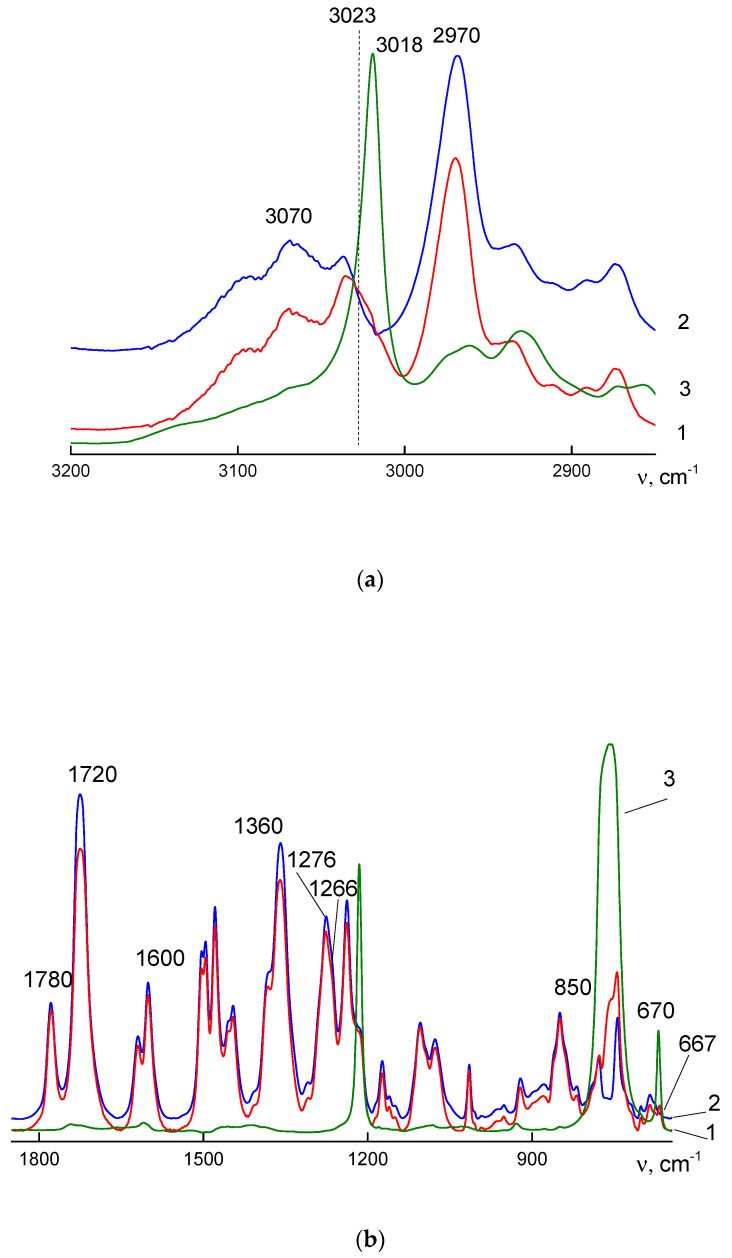
IR-spectra for films of Ultem before (curve *1*) and after ethanol treatment (curve *2*) at CH-stretching vibrations absorption region (**a**) and at “fingerprinting” region (**b**) in comparison with CHCl_3_ spectrum (curve *3*).

In addition to main absorption bands of Ultem, absorption bands of chloroform (with maxima at 667, 754 and with “shoulder curve” at 3023 cm^−1^) are registered in IR-spectra of as cast film (Figure 2). It should be noted that positions of these absorption bands maximums differ from its positions in the IR spectrum of chloroform (curve *3*, Figure 2: 670, 760, and 3018 cm^−1^), which shows a presence of noncovalent bonds between chloroform proton and PI elementary unit atom (or atoms).

Only polyimide absorption bands are registered in the IR spectra of Ultem films after all treatment methods. Figure 2 shows example of IR spectrum fragment for Ultem film after its ethanol treatment. Let us observe what happens to the conformational composition of the polyimide elementary unit.

Comparison of the IR spectra of films pre- and post-treatment did not reveal any changes in the positions of the absorption bands maxima, but differences in the relative intensity of only some of the absorption bands of Ultem functional groups were registered. For clarity, the spectra are located on the same baseline. In order to correctly compare these changes, one needs to normalize the spectrum to an “internal standard”.

**Figure 3:**
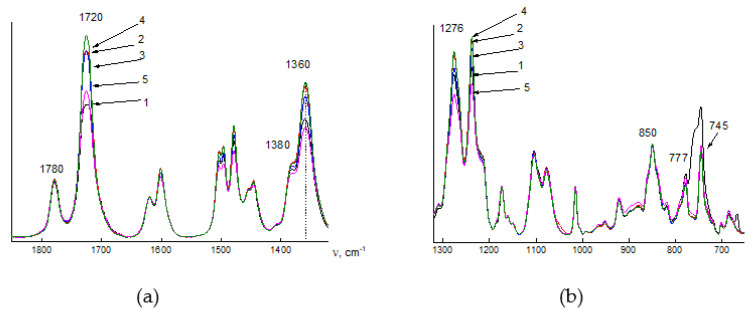
Fragments of the IR spectra of Ultem films in the region of absorption of stretching vibrations of imide and aromatic cycles (**a**) and the region of absorption of vibrations of C-O bonds, normalized to the absorption band at 850 cm^−1^ (**b**): *1*—a film formed from CHCl_3_ (as cast); *2*—as-cast film after ethanol treatment; *3*—as-cast film after *sc*-CO_2_ treatment; *4*—as-cast film after stepwise annealing; *5*—as-cast film after stepwise annealing and *sc*-CO_2_ treatment.

It can be noted that the absorption bands of some types of vibrations are completely identical in the IR spectra of films pre- and post-treatment; for clarity, all the spectra are placed on the same baseline. Given that the symmetry of the *para*-substituted phenyl rings in Ultem ensures that their spectral characteristics (maximum position, half-width, and intensity) remain unchanged regardless of the noncovalent interaction of neighboring atoms, the absorption band of deformational oscillations of neighboring H atoms during *para*-substitution in the aromatic ring (850 cm^−1^) does not change its intensity for all of film treatment methods, and it can be used as an internal standard (Figure 3). Changes in relative intensity were observed for absorption bands of symmetrical C=O stretching vibrations and CN stretching vibrations in imide cycle (1720 cm^−1^ and 1360 cm^−1^, respectively); C-O vibrations in phenyl-ether fragment (1300–1040 cm^−1^); basic vibrations of C=C-bonds in aromatic rings with maxima at 1600, 1500, and 1480 cm^−1^; and deformational oscillations of δ_CCH_ and δ_CNC_ (777 and 745 cm^−1^, respectively) [26].

Conformational changes of C–O–C bonds due to the formation or rupture of hydrogen bonds with the chloroform from which the films were formed will necessarily affect the polarization of C-H bonds in the methyl group. It would not lead to change in geometric parameters of isopropylidene group (with absorption bands maximum shift), but it will affect absorption bands intensity, which is indeed seen in spectra at 2970 cm^−1^ (Figure 2) and 1380 cm^−1^ (Figure 2 and Figure 3).

It can be concluded that all of the treatment methods of Ultem films (annealing, ethanol, and *sc*-CO_2_ treatment) may lead to conformational changes of macromolecules due to bond polarization change and conformational changes of elementary unit, and it explains difference in film IR-spectra after treatment.

Evaluation of these changes in Ultem structure and their accurate correlation with film macroparameters changes the required selection of absorption bands for which variations of spectral characteristics after treatment exceed single deviations from average values in parallel determinations (s_r_).

Moreover, changes in the structural criterion for various types of film processing should be significant. To select the structural criterion, the reproducibility of the optical density (A) measurement results was evaluated for 11 initial films and for two parallel measurements of each type of film treatment.

For values of A for which the value of the relative standard deviation from the mean ${\bar{s}}_{r}$ did not exceed 0.04 and the confidence interval ∆A was ≤ 0.07, the significance of changes in the relative optical density (A_1_/A_2_) using the modified Student’s t-test for all films was evaluated. Changes were considered significant if, according to the condition for evaluating the significance of the results for homogeneous variances, test statistics *ξ* > *t* (*P*, *f*), where *t* is the Student coefficient for the confidence probability *P* and the number of degrees of freedom *f*. For *ξ < t (P*, *f*), the changes were considered insignificant and this ratio A_1_/A_2_ was excluded from consideration as a structural criterion.

**Figure 4:**
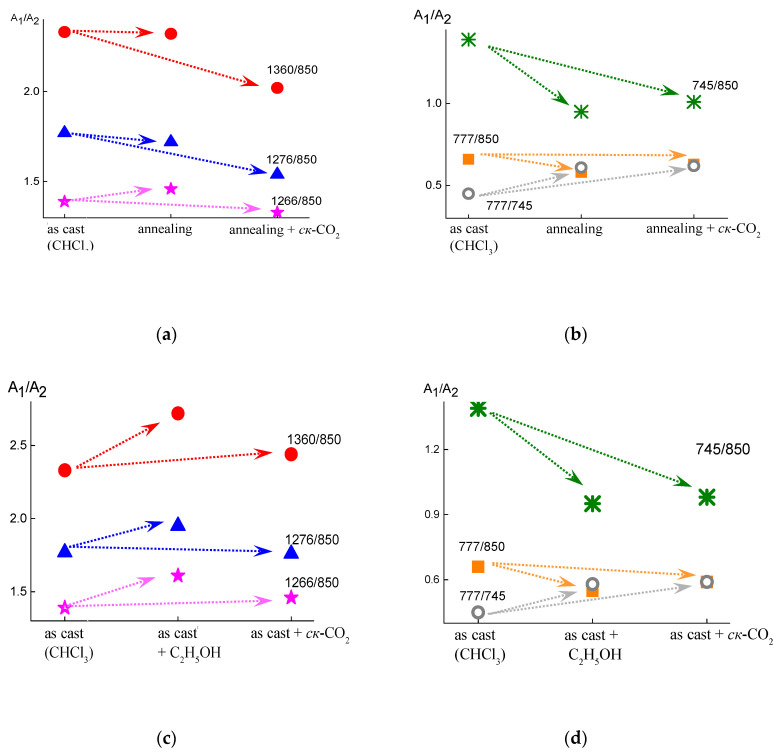
Changes in the relative intensities of the absorption bands of PI II functional groups (**a**,**c**): CO vibrations in benzimide and Ph–O–h′ groups; (**b**,**d**)—vibrations in the Im–Ph–Im′ site) depending on the processing of its films: (**a**,**b**)—as cast film, as-cast film after stepwise annealing, as-cast film after stepwise annealing and *sc*-CO_2_ treatment; (**c**,**d**)—as-cast film; as cast film after ethanol treatment; as-cast film after *sc*-CO_2_ treatment.

In Figure 4, significant changes in some structural criterion for different methods of film treatment relative to the initial film: annealing, annealing + *sc*-CO_2_ (Figure 4a,b) and ethanol treatment, and *sc*-CO_2_ treatment without annealing (Figure 4c,d) are shown.

The intensities of the absorption bands corresponding to the stretching vibrations and deformational oscillations of all CO-groups change in case of annealing of the films, which leads to desorption of chloroform, rupture of the hydrogen bonds “chloroform–oxygen” between the residual solvent and the polymer, and conformational disordering in the Ultem elementary unit.

The intensity of the absorption band decreases at 1276 cm^−1^ and the intensity of the absorption band increases at 1266 cm^−1^, which is consistent with the data in [14].

The change in the intensity of absorption bands related to the vibrations of the imide cycle, including those involving C=O groups (1720, 1360 cm^−1^), is insignificant (Figure 4a). *sc*-CO_2_ treatment of the annealed film leads to the decrease in the intensity of all these absorption bands.

On the contrary, the intensity of the absorption bands related to the vibrations of the imide and aromatic cycles in Im–Ph–Im′ unit demonstrates significant changes for annealed film, and insignificant changes for annealed film treated by *sc*-CO_2_ (Figure 4b).

Ethanol treatment of as-cast film, accompanied by desorption of chloroform, but not leading to disordering in Ph–O–Ph′ fragment, on the contrary, results in sharp increase of the intensity of the absorption bands of the corresponding vibrations (Figure 4c), while *sc*-CO_2_ treatment of as-cast film leads to insignificant changes compared to the values for as cast film. For the absorption bands of the Im–Ph–Im′ unit both ethanol and *sc*-CO_2_ treatment methods lead to significant changes in the intensity relative to as-cast film. It is interesting to note that for the absorption bands that characterize the deformational oscillations of δ_CNC_ in the imide cycle (745 cm^−1^) and δ_CCH_ in the aromatic ring between the imide cycles (777 cm^−1^), the dependences are antibate (Figure 4d). This demonstrates that Ultem film treatment affects Ph–p–Ph′ and Im–Ph–Im′ units differently and indicates the possibility of at least two fundamentally different conformational changes in pH–O–Ph′ and Im–Ph–Im′ fragments in the Ultem macromolecule depending on the treatment conditions of its films. Competing processes also explain the significant change in the s_r_ value that characterizes the reproducibility of measurement results, depending on the conditions of films treatment: the worst results reproducibility for all absorption bands was recorded for annealed films. They are characterized by the greatest conformational disordering.

In contrast to the previously studied polyimides [13], for Ultem the probability of hydrogen bonds is realized not only with the etheric O atoms, but also with the O atoms of the imide cycle, as evidenced by the change in the ratio of the ν_s_/ν_as_ intensities of the stretching vibrations of C=O and the behavior of the absorption bands at 1480 and 1360 cm^−1^, related to δ_Ar_ and δ_OCN_, respectively.

The decrease in the intensity of the absorption band at 1276 cm^−1^ (A_1276_/A_850_) and the increase in the intensity of the absorption band at 1266 cm^−1^ (A_1266_/A_850_) are corresponding to conformational disordering at the Ph–O–Ph′ site of elementary unit PI II (chloroform desorption, H-bonds destruction between the residual solvent and the polymer).

The ratio A_1276_/A_1266_ characterizes the ratio of conformers, that is, conformation order. The decrease in the intensity of the absorption bands related to vibrations of the aromatic cycle in the Im–Ph–Im′ fragment indicates the decrease in the interplanar angle due to the intermolecular π–π interaction. The ratio of intensities A_777_/A_745_ characterizes the position of the aromatic ring between the imide cycles relative to the main PI chain, i.e., intermolecular π–π interaction.

### 3.4. The Influence of the Conformation of Macromolecules on the Parameters of Packing Ordering

Suggested above as a structural criterion, the ratios of absorption band intensities of some functional groups in Ultem for samples subjected to various treatment methods can be compared with the quantitative characteristics of the packing ordering based on the analysis of gas diffusion coefficients [12] for film samples prepared under the same conditions (Table 1). The results are shown in Table 3.

It can be noted that when films are annealed (“as cast → annealing”), the selectivity of diffusion decreases by ~30%, which is accompanied by an increase of the value of A_777_/A_745_ (changes in the Im–Ph–Im′ units due to π–π interaction) by almost 30% of the value for the as cast film. Simultaneously, the ratio A_1276_/A_1266_ (change of conformational composition at the Ph–O–Ph′ fragment, disordering and increase of the content of the second conformer) decreases by approximately 10% and correlates with change in the values of Δ*S*^0^ and Δ*E_coh_*.

When the annealed film is *sc*-CO_2_-treated (annealing → annealing+*sc*-CO_2_), the diffusion selectivity α*^D^*(CO_2_/CH_4_), as well as the values of Δ*S*^0^ and Δ*E_coh_* grow symbiotically with the growth of the ratio A_777_/A_745_, and the parameter A_1276_/A_1266_ decreases by ~10%. In other words, when the annealed film is *sc*-CO_2_-treated, the crucial role in changing of the ordering parameters is played by π–π interactions, i.e., local compaction of the film in Im–Ph–Im′ units. Conformational disordering in the Ph–O–Ph′ units is accompanied by a decrease in the film density, i.e., local disordering in these fragments determines the loosening of the packing as a whole.

Ethanol or *sc*-CO_2_ treatment of as-cast film, as in the previous case, increases the probability of π–π interaction, which is accompanied by an increase in the value of the structural parameter A_777_/A_745_ relative to the initial value by ~20%; the cohesion energy, the value of Δ*S*^0^, and the selectivity of diffusion of CO_2_/CH_4_ pair of gases also increase, significantly more than for the annealed film. In this case the conformation order is maintained (the value of the parameter A_1276_/A_1266_is slightly reduced within the measurement error). Thus, noncovalent interactions with chloroform during the formation of a film from a solution set the conformational ordering in the Ph–O–Ph′ units for the Ultem macromolecule. Ethanol and *sc*-CO_2_ treatment does not violate this ordering, but contributes to additional ordering in the Im–Ph–Im′ fragments due to π–π interaction, which leads to a sharp increase in the ordering parameters (α*^D^*, Δ*S*^0^ and Δ*E_coh_*).

Analysis of these changes leads to a single explanation of these facts in total. Films treatment may lead both to some conformation ordering, especially due to hydrogen bonds with chloroform and ethanol, and to disordering, though not necessarily leading to intermolecular interaction. In Ph–O–Ph′ fragment the ratio A_1276_/A_1266_ can be considered as a structural criterion, which characterizes the ratio of conformers in this fragment, i.e., conformational ordering.

Intermolecular *π*–*π* interaction is only possible in Ultem between Im–Ph–Im′ fragments. As a structural criterion for evaluation of such interactions, we can use the intensity ratio A_777_/A_745_, which characterize the position of the aromatic ring between the imide cycles relative to the PI backbone.

**Figure 5:**
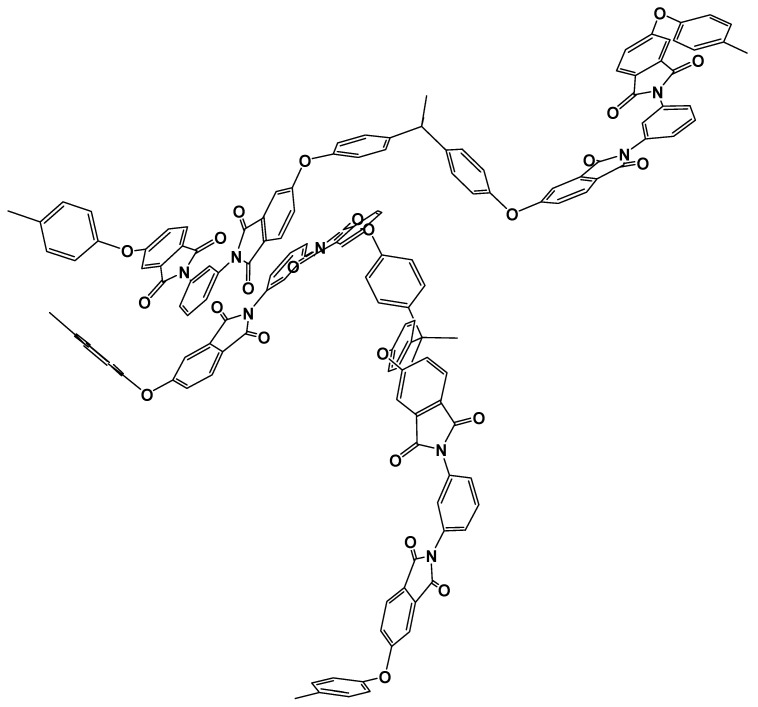
Potential relative position of PI fragments responsible for the presence of *π*–*π* interaction between Im–Ph–Im′ fragments and an increase of inter-chain distance between Ph–O–Ph′ fragments.

Experimentally observed changes in the intensity of absorption bands of only particular functional groups in IR spectra, depending on the film treatment method, can only have place if Im–Ph–Im′ fragments drawing closer simultaneously with Ph–O–Ph′ fragments distancing from each other (Figure 5). The process which will prevail depending on the Ultem film treatment type will determine the effect of conformational changes on the film properties.

Several important conclusions can be drawn from the results of this work. Treatment of films of amorphous glassy polymers containing functional groups that cause the existence of various geometric conformers in the elementary unit affects the conformational structure of the elementary unit, the conformation of the macromolecule as a whole, and the packing of polymer chains in the film. Changes of the conformational composition of a macromolecule due to noncovalent interactions of various nature are not only reflected in the vibrational spectra of a polymer object, but also correlate with the physicochemical characteristics of this object.

Practically, this effect is expressed in the possibility of explaining the seemingly illogical changes in gas transport characteristics when treating Ultem films with ethanol and *sc*-CO_2_ [12]: the film density decreases and the free volume increases, but the diffusion coefficients decrease, which cannot be explained in the framework of the free volume theory. Taking into account the results of quantum chemical calculations and comparative analysis of changes in structural criteria and quantitative characteristics of packing ordering, it can be stated that in Ultem, depending on the treatment method, both processes of ordering of the conformational structure in the Ph–O–Ph′ unit, accompanied by a local increase of the distance between macromolecules in these fragments (loosening of the package), and intermolecular π–π interaction over Im–Ph–Im′ fragments, accompanied by a local packing compaction (Figure 5) occur.

These results demonstrate that the IR spectroscopy method can be used as a tool for describing subtle structural changes that are important in the study of the structure–property relationship, and the parameters obtained from the analysis of spectra can correlate with the macroparameters of polymer objects, explaining, in particular, changes in the physicochemical characteristics of polymer films. Therefore, one should not only be guided by proper interpretation of absorption bands, but also have statistical data of spectral measurements interpretation.

## 4. Conclusions

The combination of experimental and theoretical methods of vibrational spectroscopy allows resolving of inverse problems applied to polymers to adequately identify changes of macromolecules conformational composition depending on the films treatment. The possibility of structural criterion (ratio of functional groups absorption bands intensities) selection to adequately reflect all of the conformational changes has been demonstrated and statistical processing of the measurement results confirmed accuracy of the data obtained.

For amorphous glassy polyetherimide Ultem conformational ordering of macromolecules changes depending on film treatment procedure takes place, which is verified by data of Ultem films gas separation parameters study. Moreover, the presence of residual chloroform in films favors conformational ordering of macromolecules to remain unchanged in fragments Ph–O–Ph′. The desorption of residual chloroform with ethanol or *sc*-CO_2_ leads to an increase in conformational ordering in Im–Ph–Im′ units. This can result in approximation of these chain fragments of surrounding macromolecules and in inter-chain π–π interaction (local densification of packing). The annealed Ultem film shows the most disordered (amorphous) state at all of the fragments of macromolecules.

Results of vibrational spectroscopy are well in agreement with the data of analysis of films gas separation parameters. It can be suggested that plasticization of polymer with ethanol or *sc*-CO_2_ leads to the formation of conformationally ordered parts in the polymer matrix, which maintain in a film and ensures high diffusion selectivity.

## Figures and Tables

**Table 1:** Density, fractional free volume, diffusion coefficients, diffusion selectivity, and relative changes in the parameters *a* and *b*, changes of entropy of the basic state and cohesion energy for Ultem films.

Sample, Treatment	ρ, g/cm^3^	FFV, %	D·10^8^, cm^2^/S		A*^d^*		Δ*a*, %	Δ*b*, %	Δ*S*^0^, J/mol·K	Δ*E_coh_*, kJ/mol	Ref
			O_2_	CO_2_	O_2_/N_2_	CO_2_/CH_4_					
as cast	1.294	11.2	1.5	0.33	4.4	3.6	40	24	32	50	[12,19]
as cast + C_2_H_5_OH	1.282	12.0	0.99	0.26	4.5	7.2	64	48	51	100	[12,19]
as cast + *sc*-CO_2_ *	1.276	12.4	0.46	0.11	4.1	7.6	65	55	52	115	[12,18]
as cast + *sc*-CO_2_ **	1.268	13.0	0.69	0.17	6.3	6.1	54	43	43	90	This work
annealing	1.288	11.6	0.42	0.10	4.2	2.4	0	0	0	0	[12,22]
annealing + *sc*-CO_2_ *	1.278	12.3	0.42	0.11	5.7	13.0	85	74	68	155	[12,18]
annealing + *sc*-CO_2_ **	1.268	13.0	0.67	0.13	5.2	4.3	47	37	37	78	This work

* Depressurization rate of 0.025 MPa/min, ** Depressurization rate of 45 MPa/min.

**Table 2:** Geometric and energy characteristics of two Im–Ph–Im′ fragment conformers.

Conformer	Structure	−ΔE_min_, kJ/mol	Dihedral Angle N(3)-C(6)-N(7)-O(9), Deg.	Bond Length, Å	Full Mulliken Atomic Charges		
					C	N	O
I	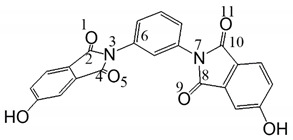	0	28.6	O(1)–C(2) 1.213 C(2)–N(3) 1.425 C(8)–O(9) 1.213 C(8)–N(7) 1.418 C(10)–O(11) 1.213	(2) 0.584 (8) 0.586 (10) 0.584	(3) 0.667 (7) 0.667	(1) 0.477 (9) 0.471 (11) 0.477
II	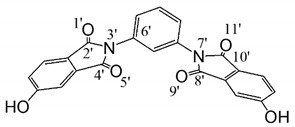	3.44 *	−34.2	O(1′)–C(2′) 1.214 C(2′)–N(3′) 1.425 C(8′)–O(9′) 1.212 C(8′)–N(7′) 1.418 C(10′)–O(11′) 1.214	(2′) 0.581 (8′) 0.582 (10′) 0.579	(3′) 0.673 (7′) 0.667	(1′) 0.478 (9′) 0.459 (11′) 0.478
III	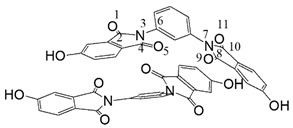	16.4 **	19.4 21.7	O(1)–C(2) 1.189 C(2)–N(3) 1.327 C(8)–O(9) 1.185 C(8′)–N(7′) 1.393 C(10)–O(11) 1.189	(2) 0.866 (8) 0.856 (10) 0.882	(3) 0.932 (7) 0.922	(1) 0.577 (9) 0.539 (11) 0.578

* Calculated as the difference between energy minima for conformers. ** Calculated as the difference between the total energy of the model and the sum of the total energies of the conformers.

**Table 3:** Quantitative characteristics of packing ordering for Ultem films and for various treatment methods based on the analysis of diffusion coefficients and structural criteria of Ultem samples prepared under equivalent conditions.

Sample, Treatment	ρ, g/cm^3^	Ordering Parameters			Structural Criteria	
		α*^D^* (CO_2_/CH_4_)	Δ*E_coh_*, kJ/mol	Δ*S*^0^, J/mol·K	A_1276_/A_1266_	A_777_/A_745_
as cast	1.298	3.6	50	32	1.27	0.48
as cast + C_2_H_5_OH	1.282	7.2	100	51	1.21	0.58
as cast + *sc*-CO_2_ *	1.268	6.1	90	43	1.21	0.59
annealing	1.288	2.4	0	0	1.18	0.61
annealing + *sc*-CO_2_ *	1.268	4.3	78	37	1.15	0.62

* Depressurization rate of 45 MPa/min.
